# Speech-to-Text Captioning and Subtitling in Schools: The Results of a SWOT Analysis

**DOI:** 10.3390/audiolres15040105

**Published:** 2025-08-12

**Authors:** Ambra Fastelli, Giulia Clignon, Daniele Corasaniti, Eva Orzan

**Affiliations:** 1 Istituto di Ricovero e Cura a Carattere Scientifico materno infantile (IRCCS) Burlo Garofolo—Audiology and ORL, Via dell’Istria, 65, 34137 Trieste, Italy; giuliaclignon@yahoo.it (G.C.); dani.corasaniti@gmail.com (D.C.); eva.orzan@burlo.trieste.it (E.O.); 2 INVALSI, Via Ippolito Nievo, 35, 00153 Roma, Italy

**Keywords:** speech-to-text, captioning, subtitling, SWOT analysis, inclusive education, hearing difficulty, education, accessibility, assistive technology, classroom acoustics

## Abstract

**Background/Objectives:** Poor classroom acoustics and inadequate digital environments in educational settings can pose an additional barrier for students, especially those with special needs, such as students with hearing difficulties. These challenges can hinder communication, academic achievement, and social inclusion. Speech-to-text captioning systems offer a promising assistive tool to support education. This study aimed to evaluate the strengths and limitations of implementing such systems in schools through a structured strategic analysis. **Methods:** The analysis method consisted of two phases. A SWOT (Strengths, Weaknesses, Opportunities, Threats) analysis was performed on data from a survey compiled by an interdisciplinary team. A subsequent TOWS analysis was used to develop strategic recommendations by cross-referencing internal and external factors. **Results:** The analysis highlighted key strengths, including improved communication, support for inclusive practices, and adaptability to diverse learning needs. Identified weaknesses included cognitive load, synchronization delays, and variability in student profiles. Opportunities included educational innovation, access to funding programs, and interdisciplinary collaboration. Threats included inadequate classroom technology, poor acoustics, and the risks of social stigma. The analysis yielded 17 recommendations to improve the usability and customization of the tool. **Conclusions:** Speech-to-text captioning systems have significant potential to promote accessibility and inclusion in education. This strategic analysis provides a structured, interdisciplinary approach to strategic planning and the successful implementation of assistive technology in schools. By combining multidisciplinary expertise with structured evaluation, it identified key design, training, and policy priorities. This approach offers a replicable model for user-centered planning and the development of assistive tools and can inform wider efforts to reduce communication barriers in inclusive education.

## 1. Introduction

Over the years, it has become established that late-diagnosed or poorly compensated pediatric hearing loss can delay or impair the development of language and comprehension skills in young children [1,2,3,4,5]. Communication difficulties increase the risk of social isolation and low self-esteem [6] and can undermine the acquisition of academic skills during school years [7,8,9,10,11,12]. The progressive advancements in clinical practice, technology, and education for children and adolescents with hearing difficulties have contributed to better academic results, with some students performing comparably to their typically developing peers [13]. However, empirical evidence still shows considerable variability within the results achieved by this group of students. Moreover, disparities between the academic performance of students with profound hearing loss and their typically developing peers tend to widen over time, becoming increasingly evident as they progress through school grades [14,15].

When hearing loss is compounded by environmental factors that hinder access to sound, such as excessive background noise or poor classroom acoustics, the consequences can become more severe. For example, listening and learning in an excessively noisy environment can disrupt speech perception, language development, and educational achievements and can be a stressful experience for all students, regardless of their hearing condition [16,17,18,19,20,21]. Noise pollution and poor environmental acoustics can have even more severe consequences for students who wear hearing aids or cochlear implants, as they hinder speech perception, increase the cognitive effort required for learning, and contribute to listening fatigue [22,23].

Unfortunately, this is often the case in school classrooms [24], so several countries—including Italy—have set up regulations to curb the problem of noise levels in school buildings and control external environmental noise, imposing valuations of the acoustic setting in new buildings and constructions in Italy [25,26]. However, most Italian school (and classroom) premises pre-date these modern regulations, often being located in buildings originally intended for other purposes that do not comply with these acoustic requirements [27]. Whilst acknowledging the importance of regulations aimed at ameliorating the school environment, it is evident that a substantial amount of work is still required.

In recent years, the measures put in place to counter the effects of the COVID-19 pandemic while guaranteeing access to the right to education and the rise of digital technology and artificial intelligence have significantly accelerated the adoption of digital tools in education. This shift has reinforced the relevance of technological solutions that enhance language access and learning. According to a focus report based on the Program for International Student Assessment (PISA)’s data from the Organization for Economic Co-operation and Development (OECD) countries, the proportion of households equipped with educational software increased from 53% before the pandemic [28] to 74% after the pandemic [29], while internet access and computer availability for schoolwork have remained consistently high over the past decade [30]. As digital learning environments have become more widespread, ensuring equitable access to information has become a pressing challenge, particularly for students with hearing loss or other learning needs. In in-person settings, access to auditory information can be improved with hearing aids [31,32] or by enhancing the signal-to-noise ratio [33,34]. However, these solutions are insufficient in remote learning environments or when the acoustic characteristics of the room are inadequate; students may struggle with poor audio quality, background noise, or a lack of visual cues. To bridge this gap, text-based support tools such as transcripts, subtitles, and captions have become increasingly relevant. A systematic review found that adding text to video improves speech intelligibility for both younger and older adults, regardless of their hearing status. Furthermore, when the auditory signal is compromised, listeners rely more heavily on text-based information to reinforce their comprehension [35]. While the use of this technology has achieved promising results in postsecondary students with hearing loss [36], the results are probably more complex for students who are still developing their reading skills, as literacy development plays a key role in how children interact with captioning. However, recommendations are recently becoming available from projects that have focused on providing guidelines for subtitling content for children with hearing loss aged 8–12 [37].

The project ABACO (*Abbattimento delle BArriere Communicative*, translated as “breaking down communication barriers”) arises from the Office for Disability Policy of the Region of Friuli Venezia Giulia and is devised and coordinated by the Audiology Department of the Burlo Garofolo Pediatric Hospital in Trieste in collaboration with the Region Friuli Venezia Giulia National Board for the Deaf and the participation of the University of Perugia and the Perugia Hospital, as well as the Fiadda Onlus in the Region of Umbria (Association for the Rights of Deaf and Hard-of-Hearing individuals). The ABACO project aims to eliminate communication barriers and promote innovative technologies and services for inclusion, education, and accessibility for individuals with hearing loss. Within this framework, an interdisciplinary group of experts conducted a series of strategic analyses to explore technological adaptations and interventions that could improve access to communication and information from school age onwards.

This paper presents the findings of a SWOT analysis (Strengths, Weaknesses, Opportunities, Threats) conducted during a meeting focused on “Speech-to-text Captioning at School: Perceptual and Educational Aspects”. The meeting aimed to define the key characteristics of an ideal speech-to-text captioning system for classroom use. In our study, “speech-to-text”, also referred to as automatic speech recognition, refers specifically to real-time captioning of spoken language during interactions or videos, intended to support accessibility for students.

Originally introduced by Learned et al. in 1969 [38] as a method for addressing complex strategic issues by condensing large amounts of information, SWOT analyses are now widely used to evaluate the internal strengths and weaknesses of products, projects, or services, as well as to identify opportunities and threats in the external environment [39,40,41]. To the best of our knowledge, this type of analytical approach has not been systematically used to evaluate speech-to-text technologies in educational settings. However, recent studies have highlighted the potential of captioning tools to support students with hearing loss by improving accessibility and learning outcomes. Advances in automatic captioning systems that utilize artificial intelligence to improve output quality have also been suggested as a way to empower users and provide personalized support [42]. Additional research has demonstrated that the non-textual properties of captions, such as speaker labels, positioning, and occlusion, play a critical role in their effectiveness from a user experience perspective [43].

In this context, the present study builds on the previous literature by using a SWOT framework to focus on a specific aspect of the broader ABACO project: the development of a speech-to-text transmission system. The system is envisioned as compatible with hearing aids and subtitling modes, is usable across different devices, and is designed to minimize the latency in subtitle generation. To contribute to the removal of communication barriers, the interdisciplinary working group examined both the perceptual and educational aspects of a potential speech-to-text captioning system in schools. The findings of this analysis are presented in the following sections.

## 2. Materials and Methods

This study was conducted using a SWOT analysis framework [38], which comprises two phases: SWOT and TOWS. The method was applied to examining both the internal factors (Strengths and Weaknesses) and external factors (Opportunities and Threats) of a speech-to-text captioning system for educational settings, in order to gain a realistic perspective on the project’s feasibility and potential. The SWOT matrix evaluates internal factors (strengths and weaknesses) and external factors (opportunities and threats) to provide a comprehensive and realistic perspective on a project’s feasibility and potential.

A TOWS analysis often follows a SWOT analysis as part of a two-phased method. While both phases use the same categories, their purpose differs: SWOT identifies and organizes key factors, whereas TOWS systematically combines these factors to generate strategic options. In short, SWOT helps identify and understand internal and external factors intervening in a subject, while TOWS focuses on developing strategies based on these insights. Internal factors are typically variables subject to the control of the organization in which one operates (e.g., school, research institute, IT company, hearing aid center, speech therapy service, family, etc.), in which it is possible to intervene through short-, medium-, or long-term programs. External factors are those dynamics over which the organization has no control nor the ability to directly intervene in (e.g., social context, legislative aspects, etc.).

This study followed a multi-phase process consisting of an interdisciplinary meeting and a discussion, a survey using a SWOT matrix, and SWOT and TOWS analyses. The online meeting included 22 participants, representing a diverse group of stakeholders: university students with hearing loss; professionals with specific roles in the prevention, diagnosis, and treatment of pediatric hearing loss; special education teachers; researchers and lecturers; and members of the public advocating for people with hearing loss. Participants were invited from within the ABACO project team. They were not recruited specifically for this study. From this existing pool, participants with both professional and non-professional backgrounds were purposefully selected by the research director to ensure representation from the various sectors involved in school inclusion and hearing loss. Although no fixed target number of participants was established beforehand, efforts were made to involve as many relevant perspectives as possible to support a robust and interdisciplinary analysis. For example, university students with hearing loss were included because they have personal experience with hearing technologies and a reflective understanding of their educational experience, enabling them to offer valuable insights into the long-term impact of accessibility tools such as captioning. Younger students (e.g., primary, middle, or high school students) were not included to avoid ethical concerns relating to minors participating in an experimental strategic meeting and because young adult participants could confidently reflect on their experiences across different educational levels. In sum, participants were selected due to their expertise in education, audiology, and/or technology or their personal experience of hearing loss and for their ability to contribute unique perspectives to a holistic evaluation of speech-to-text captioning systems in school settings. Table 1 provides an overview of the interdisciplinary team, distinguishing between the total number of participants who attended the strategic discussion meeting and those who actively contributed to the SWOT analysis by completing the survey.

The two-hour discussion focused on defining the key characteristics of an optimal speech-to-text captioning system for schools, incorporating insights from neuropsychological research, teaching strategies across different age groups, and students’ specific needs. A moderator with hearing loss facilitated the session to ensure accessibility for all participants. During the meeting, three participants were selected to frame the problem and present the current state of the art based on their experience and expertise: a primary school teacher shared her knowledge of the educational context and classroom challenges; two students with hearing loss provided their insights into accessibility needs; and a cognitive researcher contributed with his scientific knowledge on language processing and learning. Together, they balanced the representation of practical, personal, and research perspectives within the larger interdisciplinary team. The discussion unfolded in three stages: (1) identifying challenges in primary school education for students with hearing loss, (2) discussing the cognitive and attentional overload risks associated with subtitled content, and (3) exploring the ideal features of a captioning system based on personal experiences shared by university students with cochlear implants. Throughout the meeting, the participants engaged in an interdisciplinary discussion, comparing their perspectives and viewpoints after each contribution.

This meeting was one of a series of scheduled sessions within the broader ABACO project. As is standard practice, the participants were sent an email invitation containing logistical details and a general description of the topic, and they were then given a brief introduction to the objectives at the start of the meeting. To frame the research component and ensure informed participation, the study’s aims and procedures were explained in detail at the end of the meeting, and verbal consent was requested to participate. Those who agreed were invited to individually complete a survey in the form of a SWOT matrix, structured as a 2 × 2 strategic planning table covering four areas: Strengths, Weaknesses, Opportunities, and Threats (Table 2). The survey prompt was “What characteristics should an ideal school speech-to-text captioning system have, considering current neuropsychological research, the teaching needs of different age groups, and student requirements?”. Each participant was asked to provide at least two responses per category, based on their professional expertise and personal experience. The survey was non-anonymous, allowing the responses to be contextualized according to the participant’s role and profession. Participation was entirely voluntary. The respondents received a small monetary token for completing the task and submitted their responses to the project manager coordinating the study within a week.

A three-member analysis team was selected by the project manager to process the SWOT data. The team consisted of a primary teacher specialized in special needs, a psychologist with expertise in deaf studies and special education, and a person with cochlear implants, also contributing as a potential end-user.

The analysis process followed these steps: (1) *Independent Categorization:* Each team member independently reviewed the responses, categorizing each one with a representative label (key point). No prior discussion took place to ensure objective categorization. (2) *Consensus-building:* The three members of the team met to compare their findings, resolve discrepancies, and agree on a unanimous set of key points. (3) *Relevance Estimation:* Key points were quantified, with only those occurring in ≥8% of responses being included in the following step of the analysis. This threshold, while arbitrary, ensured focus on the most relevant insights.

Following the SWOT analysis, a TOWS matrix was developed to strategically align external with internal factors. The TOWS creation is not an operation of mathematical systematicity but rather a procedure that is designed to extract the recommendations and strategies that emerge from the whole strategic analysis process. The TOWS matrix reverses the SWOT matrix and confronts the key points for each area, comparing factors by pairs to formulate actionable strategies: (1) Strengths–Opportunities (S-O): Leveraging strengths to capitalize on opportunities; (2) Weaknesses–Opportunities (W-O): Addressing weaknesses by harnessing opportunities; (3) Strengths–Threats (S-T): Using strengths to mitigate external threats; and (4) Weaknesses–Threats (W-T): Minimizing weaknesses to avoid threats. The mining of the strategies follows a maxi–mini approach, as is described in Table 3. This approach facilitated the extraction of concrete recommendations, ensuring that the proposed solutions maximized the benefits while minimizing risks. The final strategic insights derived from the TOWS analysis are presented in the following section.

## 3. Results

### 3.1. Survey Participation and Data Collection

Out of the 22 initial participants, 16 completed the survey, yielding a response rate of 73%. Among the collected SWOT matrices, 14 were completed by professionals involved in the prevention, diagnosis, and treatment of pediatric hearing loss, 1 was completed by a student with hearing loss, and 1 was completed by a parent affiliated with FIADDA (the Italian Association for the Rights of Deaf and Hard-of-Hearing individuals) (Table 1). A total of 194 open-ended responses were recorded across the four SWOT categories: 50 Strengths, 51 Weaknesses, 45 Opportunities, and 48 Threats. These responses were systematically reviewed and grouped into key points (Table 4) according to the methodology described earlier.

### 3.2. The Main Key Points That Emerged from the SWOT Analysis

#### 3.2.1. Strengths

a. *Inclusive Education.* Strategies, adaptations, and tools that foster flexible and student-centered teaching, more attentive to the specific needs of each student, including those with hearing loss.
b. *Enhanced Communication.* Educational, contextual factors that improve the information exchange between teachers and students.
c. *Personalized Learning Strategies.* Tailored teaching approaches that accommodate diverse learning needs, allowing all students—including those with hearing loss—to reach their full potential, involving individualized learning, where students pursue different learning goals; personalization involves using diverse teaching strategies (e.g., varying methodologies, timeframes, and tools) to achieve the same educational objectives.
d. *Integrated-bimodal Communication.* The simultaneous engagement of auditory and visual sensory pathways to facilitate comprehension.
e. *Technology.* Already existing and potential technological resources in schools (e.g., interactive whiteboards, tablets, and Wi-Fi) that can support digital innovation and accessibility in education. Relevant national initiatives for Italy include the National Digital School Plan (PNSD) [44] and the National Operational Program (PON) by the Ministry of Education, Universities and Research.

#### 3.2.2. Weaknesses

a. *Cognitive Load and Listening Fatigue.* The mental strain resulting from keeping sustained attention and processing both auditory and visual input simultaneously and the listening fatigue that is typically experienced by students with hearing loss.
b. *Synchronization.* Challenges in maintaining accuracy and speed between audio content and transcript text.
c. *Individual Characteristics of the Student.* Variability in reading proficiency, attentional capacity, and neurocognitive development and the presence of specific difficulties (e.g., specific learning disorders) or other characteristics of vulnerability (special education needs).
d. *Background Noise.* In this context, we refer to the background noise and reverberation which may be found in classrooms that are poorly soundproofed and too crowded.
e. *Teachers’ Characteristics.* Differences in teaching styles, motivation, and willingness to engage in ongoing professional development affect the successful adoption of assistive technologies.

#### 3.2.3. Opportunities

a. *Educational Innovation.* The implementation of new teaching strategies and tools used to enhance learning environments and promote digital innovation in schools. This is particularly relevant given the objectives and funds set out by the PNSD, as well as recent advancements in e-learning.
b. *Educational and Social Inclusion.* The process of promoting inclusiveness and accessibility in school—for example, by facilitating the participation of every student with the overall aim of maximizing the learning potential of the whole class group.
c. *Advancements in Technology.* The development of new assistive technologies or the adaptation of what is already available so that they can be flexibly used in different situations (not only schools but also public offices, public administrations, railway stations, etc.) or used to improve the autonomy and integration of individuals with hearing loss.
d. *Interdisciplinary Collaboration and Synergy.* The active collaboration, communication, and exchange of perspectives between partners in the ABACO project (schools, health care, universities, associations, families, technicians, institutions, etc.) promote the integration of different types of expertise to effectively reach its aims—in this case, the development of a user-centered speech-to-text system in line with the needs of the end-users. Since the ultimate goal of a SWOT analysis is to obtain insights, multi-disciplinarity is a core value. Moreover, this synergy represents a sounding board for future diversified collaborations.

#### 3.2.4. Threats

a. *Need for Educational Activity Organization.* Certain educational activities (e.g., foreign language lessons, workshops, or laboratory work, when using physics or mathematic notations, Montessori or outdoor education approaches, etc.) may pose difficulties with subtitling systems.
b. *Lack of Technological Equipment in Schools.* Some schools may lack adequate digital equipment or internet connectivity, which could hinder the effective implementation of a speech-to-text system. This includes possible technical problems after the implementation of the system, which could negatively influence everyday practice.
c. *Usability Issues Experienced by the Student.* Some students may experience difficulties with the use of software or adapting to the subtitling system. For example, the speed of transcription or its mode (e.g., literal or synthesized transcription) may not perfectly fit the learner’s cognitive and/or linguistic abilities, their reading skills, or their preference for more emphasis on lip-reading. When these needs are not taken into account, the risk of fatigue and excessive cognitive load for the student increases.
d. *Social Stigmatization.* Customized educational tools, despite their benefits, may inadvertently contribute to stigma if they are perceived as exclusive accommodations for students with disabilities. This risk also applies to the speech-to-text system at school.
e. *Classroom Acoustic.* Common acoustic issues (e.g., excessive background noise, reverberation, poor soundproofing, overcrowded classrooms, and poor speech intelligibility) could limit the effectiveness and usability of speech-to-text technologies.

### 3.3. The Transition to the TOWS Analysis

The 19 key points identified through the SWOT analysis have been elaborated to formulate recommendations aiming to guide the development of the speech-to-text system. These recommendations were derived using the TOWS matrix (Table 3), which aligns the SWOT elements to propose strategic solutions. At least four recommendations were extracted for each cross-section, with five derived from the Strengths–Threats combination. The number of recommendations was not predetermined but rather guided by their relevance and potential impact, ensuring that each key point was addressed comprehensively while avoiding redundancy.

### 3.4. Recommendations and Strategies That Emerged from the Strategic TOWS Analysis Process

#### 3.4.1. Strength–Opportunity (S-O) Strategies

1. The integration of speech-to-text systems into teaching strategies enhances the inclusion of students with disabilities in the modern educational landscape.
2. Schools can maximize national funding opportunities (e.g., PNSD and PON) by adopting this innovative assistive technology.
3. The use of advanced, multi-functional technologies extends beyond the classroom, fostering broader social inclusion in various public settings (e.g., schools, train stations, courts, and hospitals). This is particularly beneficial in smaller or less accessible areas where the deaf community is less represented.
4. Interdisciplinary collaboration is a strength for implementing a user-centered system aligned with their needs. In addition, this synergy fosters future cooperative projects across different fields.

#### 3.4.2. Weakness–Opportunity (W-O) Strategies

1. Cognitive load and listening fatigue can be mitigated through two complementary approaches: (a) the continuous refinement of assistive technology, and (b) the integration of technology into teaching practice by trained and motivated teachers. As teaching and technological tools become more responsive to students’ needs, their cognitive load and fatigue decrease.
2. Transcription and synchronization problems can be progressively reduced through advancements in speech-to-text technology and active collaboration between developers, educators, and users.
3. Raising awareness of background noise inside the class can help to minimize it. This may require specific training on acoustic management; however, it would not only improve the learning environment for students with hearing loss but also benefit the entire class.
4. Recognizing and addressing individual student needs should not be seen as an obstacle but rather as an opportunity to refine inclusive teaching practices. This opportunity can be maximized through teacher training, as well as collaboration between experts and technicians.

#### 3.4.3. Strength–Threat (S-T) Strategies

1. Effective communication between teachers, students, and specialists can facilitate the planning of daily and weekly learning activities and the related use of speech-to-text, expanding the support available to students and teachers.
2. The presence of a student with hearing loss in a school with limited technological resources can increase the institution’s eligibility for national funding programs (e.g., PNSD and PON). These funds can be used to acquire assistive technology, which will become part of the school’s equipment available to all students with special needs.
3. The need related to the use of speech-to-text and the training that supports this project can contribute to raising awareness about the acoustic problems at school and encourage forwarding requests to municipal administrations for compliance with the law about the levels of sound in school environments.
4. Greater awareness of acoustics-related challenges can lead to environmental modifications that benefit the entire school community.
5. The interdisciplinary approach in this project supports both educational personalization and technological adaptation, ensuring that the system meets the unique needs of each user.

#### 3.4.4. Weakness–Threat (W-T) Strategies

1. The challenges associated with speech-to-text usage in particular teaching situations and those related to the risk of cognitive load or listening fatigue could be mitigated by applying technical improvements (e.g., better synchronization, pre-loading symbols and formulae, etc.) and/or using educational strategies (e.g., teacher training, structured teaching planning, etc.).
2. Technical issues can be minimized by deploying systems capable of operating on local networks, thereby enhancing synchronization, transcription speed, and reliability in parallel.
3. Background noise reduction can be achieved by improving classroom acoustics, in compliance with the current regulations, and through specific training of professionals working in the school. This can benefit the whole school community and can be useful in other circumstances related to the individual characteristics of students (e.g., multilingual learners, etc.).
4. The risk of usability difficulties for students can be mitigated by designing a customizable system that adapts to individual preferences or profiles the preferences of the end-user (e.g., subtitling speed, transcription style, level of emphasis on lip-reading, microphone use). A trained teacher can help identify usability challenges even if the student does not explicitly express them.

## 4. Discussion

Disrupted access to sound, whether due to internal causes such as degree of hearing loss or external causes such as inadequate acoustics in school environments, can negatively impact learning outcomes; increase stress levels and social isolation; and have significant long-term effects on academic and interpersonal achievement. Although these effects are more pronounced on people with special educational needs, the negative consequences may also impact on the entire class, including teachers. Therefore, promptly and effectively eliminating any barriers to understanding and communication in the school environment should be a primary and urgent goal for both schools and modern society.

Strategic analyses were carried out to systematically examine the most relevant aspects, both beneficial and challenging, regarding the implementation of an innovative technological system for translating and enhancing communication at school. The interdisciplinary strategic analysis allowed the identification of key aspects with potential benefits or risks for achieving this objective. By analyzing and discussing the data obtained through this process, a total of 19 key points were identified, from which 17 recommendations and strategies were drawn. Rather than serving as a conclusion, these recommendations mark a strategic starting point for addressing the actual needs of students with hearing loss and their teachers, optimizing resources, and facilitating the achievement of the goals.

Within this framework, three main aspects were identified, which may contribute to the effective implementation of a speech-to-text system for schools. If successful, this project could also lead to the extension of the speech-to-text system to other contexts. These aspects are as follows:

1. The interdisciplinary discussion highlighted the need for tools tailored to the individual characteristics and specific needs of students with hearing loss.
2. The use of technological devices and systems in schools represents a viable opportunity for educational innovation and access to dedicated funding and resources. Moreover, the system provides valuable support for all students, not only those with hearing loss or special education needs thanks to its customizable features.
3. Implementing a technologically advanced and cost-effective tool not only encourages its use but also contributes to identifying new adaptations and innovative educational strategies (i.e., integrating the tool into daily practice). Additionally, the system offers teachers opportunities for professional development, covering both the technical aspects of system usage and the specific educational needs of students with hearing loss.

The interdisciplinary meeting of the study group facilitated a constructive and collaborative discussion, allowing each participant to independently identify the most relevant aspects from their disciplinary perspective or personal experience. Based on the information gathered, and through the strategic analysis, it was possible to formulate recommendations that clearly defined key aspects that, although shared by various group members, had not been systematically and explicitly articulated before. The primary advantage of this approach is that the system developed based on these recommendations is more likely to align with end-users’ needs, thereby potentially increasing the likelihood of successful classroom implementation (or beyond). Additionally, this approach may support the more effective allocation of resources by providing a clearer basis for targeted planning and funding decisions.

The small number of participants in the initial study group represents a potential limitation to this analysis. However, the group encompassed a wide range of professional and experiential profiles, and the limited number of participants somehow encouraged active and equitable discussion, ensuring that each participant had the opportunity to express their unique perspective on the strengths and weaknesses of the project. While the small group size led to a limited number of responses in the SWOT analysis, the number of participants generally exceeded the recommended minimum of two responses per area, suggesting a high level of engagement, likely supported by the structured format and prior thematic discussion (average number of responses per SWOT area: S = 2, 94; W = 3, 06; O = 2, 88, T = 3, 19). A further limitation is that the analysis relied on participants’ expertise and professional backgrounds rather than on direct case studies or observational data. Therefore, this analysis does not rule out the possibility that unanticipated challenges or opportunities may emerge during the system’s actual implementation or real-world use. Future research should incorporate real-world trials and involve a broader range of professionals and individuals from various stages of education (e.g., primary and secondary school) to ensure the solutions respond to the diverse needs and contexts of all learners.

## 5. Conclusions

In summary, this investigation illustrated that a strategic analysis enabled the effective exchange of information within the working group. For example, professionals specialized in one discipline (e.g., healthcare) were facilitated to explore the aspects of other sectors (e.g., classroom acoustics). Actively engaging in this interdisciplinary process broadened the participants’ perspectives and enhanced their critical analysis skills. This approach also allowed the participants to proceed with the later stages of the project with a more structured and profound understanding. In this regard, the use of a strategic analysis during the initial project phase likely optimized resources both in terms of time and financial investment in later stages.

Overall, this analysis supports the need for and the potential benefits of implementing a speech-to-text system in schools. Ideally, this system will catalyze further advancements in technological and educational innovation, helping to eliminate communication barriers. Adopting this innovative system may also facilitate access to funding for digital education initiatives, which many countries—including Italy—have established to support digital innovation. Given the architectural situation of many school classrooms, fully resolving the acoustic problems that are currently present would require a great deal of time and funding. In the meantime, a tool that eases the cognitive load and listening fatigue could be an indispensable support for students with special needs and useful for everyone. To maximize the effectiveness of the subtitling program, it is necessary for it to be customizable according to the needs of students and teachers. Thus, conducting a strategic analysis with professionals and end-users who identify the potential strengths and critical challenges of the program is an excellent way to maximize its success rate.

## Figures and Tables

**Table 1:** A description of the participants (the interdisciplinary team) involved in the survey.

Participants’ Role	Nr. Meeting Participants ^1^	Nr. of Participants Who Completed the SWOT Matrix ^2^
Otolaryngologists or physicians in audiology	3	3
Audiologists or hearing acousticians	2	1
Speech and language therapists	2	2
Psychologists	1	1
Psycholinguists	1	0
University lecturer (cognitive science)	1	1
Researcher (cognitive science)	1	1
Computer scientists or sound engineers	3	2
Primary teacher for children with special needs	1	1
Secondary school teacher	1	1
School inclusion expert (regional school office for FVG ^3^)	1	1
Members of the FIADDA (association of parents of deaf children)	2	1
University students with hearing loss	2	1
Total	22	16 (73%)

^1^ Number of participants who attended the meeting for the strategical analysis; ^2^ number of participants who contributed to the SWOT (Strengths, Weaknesses, Opportunities, Threats) analysis; ^3^ Friuli-Venezia Giulia.

**Table 2:** A SWOT matrix of the questions used to help with the identification of strengths, weaknesses, opportunities, and threats.

	Helpful for the Objective	Harmful to the Objective
**Internal factors**	Strengths (S)	Weaknesses (W)
	What do we do well? What advantages do we have? What relevant resources do we have access to? What do others see as our strengths?	What could be improved? Where are fewer resources placed? What is perceived by others as a weakness? What should we avoid?
**External factors**	Opportunities (O)	Threats (T)
	What opportunities are opening? Which trends should be taken advantage of? How to turn strengths into opportunities?	What are the risks/impediments that might hinder us? What are other groups, organizations, and companies doing? What risks do our weaknesses expose us to?

**Table 3:** TOWS matrix concept (maxi–mini approach).

		*Internal Factors*	
		Strengths (S)	Weakness (W)
*External factors*	**Opportunities (O)**	(S-O)	(W-O)
		Maxi–maxi strategy. Strategies that use strengths to maximize opportunities.	Mini–maxi strategy. Strategies that minimize weaknesses by taking advantage of opportunities.
	**Threats (T)**	(S-T)	(W-T)
		Maxi–mini strategy. Strategies that use strengths to minimize threats.	Mini–mini strategy. Strategies that minimize weakness and avoid threats.

**Table 4:** Key points identified by the SWOT analysis.

SWOT Areas	Key Points	*N* (%)
**Strengths**	Inclusive education	15 (30%)
	Enhanced communication	9 (18%)
	Personalized learning strategies	8 (16%)
	Integrated-bimodal communication	5 (10%)
	Technology	4 (8%) ^1^
	Teachers’ motivation and continuing education	3 (6%)
	Individual student characteristics	2 (4%)
	Personal autonomy	2 (4%)
	Collaboration	1 (2%)
	School access to financial resources	1 (2%)
**Weaknesses**	Cognitive load/listening fatigue	15 (29, 41%)
	Synchronization	8 (15, 68%)
	Individual characteristics of the student	7 (13, 72%)
	Background noise	6 (11, 76%)
	Teachers’ characteristics	5 (9, 80%) ^1^
	System accuracy	4 (7, 84%)
	Poor versatility	3 (5, 88%)
	Stigmatization	2 (3, 92%)
	Technology bounded	1 (1, 96%)
**Opportunities**	Educational innovation	16 (35, 55%)
	Educational and social inclusion	8 (17, 77%)
	Advancement in technology	6 (13, 33%)
	Interdisciplinary collaboration and synergy	5 (11, 11%) ^1^
	Teachers’ motivation and continuing education	3 (6, 66%)
	Learning opportunity	3 (6, 66%)
	Communication	2 (4, 44%)
	Personal autonomy	2 (4, 44%)
**Threats**	Need for educational activity organization	10 (20, 83%)
	Lack of technological equipment in schools	9 (18, 75%)
	Usability issues experienced by the student	6 (12, 5%)
	Social stigmatization	5 (10, 4%)
	Classroom acoustics	4 (8, 3%) ^1^
	Poor accessibility of technical support	3 (6, 25%)
	Teachers’ poor motivation	3 (6, 25%)
	Teachers’ lack of specific training	3 (6, 25%)
	Nonacceptance	3 (6, 25%)
	Synchronization	1 (2%)
	Unintelligibility due to the mask barrier	1 (2%)

^1^ Only the key points that exceeded the 8% threshold were considered for the TOWS analysis. This threshold is arbitrary and simply based on a principle of relevance.

## Data Availability

The data supporting the findings of this study (non-anonymous survey) are not publicly available due to privacy and ethical restrictions.

## Abbreviations

The following abbreviations are used in this manuscript:

SWOT	Strengths, Weaknesses, Opportunities, Threats
TOWS	Threats, Opportunities, Weaknesses, Strengths
PISA	Program for International Student Assessment
OECD	Organization for Economic Co-operation and Development
ABACO	Abbattimento delle BArriere Communicative, translated as “breaking down communication barriers”
